# Travellers’ Health: How to Stay Healthy Abroad, 4th edition

**DOI:** 10.3201/eid0904.030005

**Published:** 2003-04

**Authors:** Peter J. Baxter

**Affiliations:** University of Cambridge, Cambridge, U.K.

Since the first edition of this book was published in 1986, travel medicine has flourished as a specialty, with seemingly no end to the expansion of air travel and the number of intrepid persons seeking out remote destinations for work or pleasure. The original edition was immediately received as the best general guide available for health professionals who dispense advice and medications to travelers, as well as for travelers who want more information about potential hazards they might encounter. This fourth edition, which is even more comprehensive and authoritative than its predecessors, continues the original successful formula and can be unreservedly recommended to anyone, expert or nonexpert, interested in the subject. Almost every conceivable topic is covered in the 75 chapters, each written by an expert or group of experts drawn mostly from the United Kingdom, with enough contributors from the United States to provide an international dimension.

One of the most useful sections, for travelers at higher risk, focuses on those who are pregnant, very young, elderly, and disabled, as well as those with HIV infection. Medical practitioners will find common topics addressed for patients with chronic, even life-threatening, conditions who ask about their fitness to travel and receive immunizations. Expedition health and medical kits are also well covered, and many chapters have references or guidance on accessing more information. A variety of sporting and recreational activities are discussed in individual chapters. Even humanitarian workers are considered with the inclusion of a chapter on land mines. Expatriates get a full discussion, including a warning not to habitually complain about their servants to expatriate colleagues, as well as a valuable section on personal security. Otherwise, the text follows the familiar disease and disease vector chapter format with excellent clarity and conciseness.

Acceptable online services supply up-to-date information for travelers on country-by-country health hazards, but none can compete with Dawood’s guide. This gold mine of information is a fascinating read for even the armchair explorer. Travellers’ Health is the authoritative guide for regular travelers, especially for anyone planning to live and work in foreign climes. For the health professional, the volume also serves as an accessible compendium of information about unfamiliar infectious diseases and environmental hazards and their prevention. And, despite the thoroughness of its content, the book has remained slim enough to pack away in the carry-on flight bag.

